# Patient-Reported Outcome Measures in Diseases of the Head and Neck

**DOI:** 10.3390/jcm11123358

**Published:** 2022-06-11

**Authors:** Athanasia Printza

**Affiliations:** First Otolaryngology Department, Medical School, Faculty of Health Sciences, Aristotle University of Thessaloniki, 54124 Thessaloniki, Greece; aprintza@auth.gr

Patient-reported outcome measures (PROMs) are measurement tools that capture a person’s perception of their own health. A patient-reported outcome (PRO) is any report of the status of a patient’s health condition that comes directly from the patient. Health outcomes assessment is a crucial part of health care. A health outcome is a change in the health status of an individual, a group of people, or a population, which results from measures or specific health care interventions regardless of whether such an intervention was intended to change their health status (WHO, 1998). Principal forms of health outcomes are measures of physiological parameters (biomedical indicators), clinicians’ ratings of their patients’ health outcomes, and the routine collection of outcome-related indicators by healthcare organizations. Patient-reported outcomes encompass a wide range of measurable outcomes of care from the patient’s perspective, including symptoms, functional status, and health-related quality of life (HRQoL) [1]. A PRO is directly reported by the patient without the interpretation of the patient’s response by a clinician or anyone else [2].

Patient-Reported Outcome Measures (PROMs) are the instruments used to measure PROs. The patients are asked to complete standardized validated questionnaires so that they self-assess their own symptoms, wellbeing, and functional status and rate their health by responding to a series of items/questions, which are then combined to represent an underlying construct such as symptom severity, function, or quality of life. Self-reported outcomes may correlate poorly with other outcomes such as biomarkers, clinician-reported outcomes, or performance-related outcomes. PROMs value patients as experts on their experiences [3]. The growth in the academic literature on PROMs reflects a growing global recognition that incorporating the patient’s perspective is integral to the quality and effectiveness of health care.

PROMs enable patients to provide information on aspects of their health status that are relevant to their quality of life, including symptoms and daily functioning and physical, mental, and social wellbeing [4]. Often, generic (applied across different populations) and disease-specific (used to assess outcomes that are specific to a particular disease or sector of care) PROMs are administered at the same time as they provide complementary information, since generic tools are likely to lack sensitivity to capture outcomes related to diseases of the head and neck [4].

PROMs can facilitate the tracking of health outcomes over time, enable comparisons between a patient’s outcomes and those of other patients with the same health conditions; enhance the provision of person-centered care, and contribute to value-based care; improve clinician–patient communication; inform shared decision making; and be part of the analysis of the comparative effectiveness of treatments, variations in care, costs, and outcomes among healthcare providers and the effectiveness of the implementation of quality improvement activities. Therefore, PROMs can be used by patients and clinicians (to inform clinical care, to improve patient–provider communication, and patient involvement in decision-making), researchers and policy-makers (to conduct comparative effectiveness analyses and answer cost-effectiveness questions), and health system decision-makers (to inform health service planning and policies, for performance measurement, and quality improvement initiatives).

Alongside their use in clinical settings and in organizational performance measurement, PROMs have applications in government policy making. The cost-effectiveness of different healthcare interventions needs to be determined when deciding on resource allocations and best clinical practice. Such policy decision making cannot be made only on the basis of studies conducted under highly controlled conditions, such as randomized controlled trials (RCTs). The ‘real world’ data providing evidence regarding the impacts of interventions must also be taken into account. The patient population and management approaches for any given intervention are typically more diverse than those found in RCTs. Since they capture highly relevant information, PROs are a source of ‘real world’ data used to inform healthcare decisions about effective treatments. Garrison and colleagues have described PROs as ‘the only direct voice that an individual has in the health decision-making process’. It has been suggested that PROMs can help providers promote a more personalized health system and develop tailored recommendations for screening and prevention. PROs can be used to develop models to estimate the value of particular screening tests.

Several authors have advocated for the importance of administering PROMs through the continuum of care. PROMs can provide information about baseline HRQoL at the initial visit, and can help in evaluating disease progression or regression and treatment effects at subsequent visits [5]. They provide insight into patient preferences and behaviors [3]. Collecting data from PROMs may have additional benefits by supporting patient-centered care in routine practice. Patient involvement in outcome reporting may be health-promoting, by enhancing the patients’ engagement in monitoring changes in their own condition or improving adherence. 

A key challenge for the use of PROMs is selecting a reliable and valid tool that is appropriate for one’s specific purpose from among the hundreds of instruments available [4]. A rigorously developed and validated PROM may conceivably have a varying degree of validity depending on its use in a broad range of contexts (different countries and care settings), populations (older patients and condition subtypes), and purposes of use (decision making with individual patients, provider comparison, and funding health-care organizations) [6]. The selection of which PROM to use is based on several parameters: The PROM shows high reliability (consistency of measurement), e.g., internal consistency and test/retest reliability. The PROM has high documented validity; the instrument measures what it claims to measure. There are different types of validity: content, construct, criterion, concurrent, convergent, discriminant, etc. The PROM scales and scores should have high content and construct validity (structural, responsiveness, convergent, and predictive). The PROM has been shown to be able to discriminate well between groups, for example, healthy people versus people with disease, and can detect changes in health status over time. Comparative data, mainly norms and clinical reference datasets, are available for comparative purposes [6]. The PROM scores are easily interpretable. Culture and age appropriateness have been documented. The PROM has a low respondent/administrative burden (the process of data collection does not place undue burden on the patients or the healthcare team). The PROM process is not too long, it is easy to administer, and the literacy level of the survey is appropriate [7]. Confounding factors should be identified. The social desirability of the responses or inappropriate questions could be associated with missing data. Different types of instruments (generic and/or disease-specific measures) and modes of administration (self-reporting, interview, telephone administration, tablet, or online kiosk application) are appropriate for different uses. A position statement released by the Mayo Clinic to educate community hospital stakeholders about the merits of collecting and reporting PROs describes a good PROM as simple (it can be read by a 12-year-old), brief (takes no more than 12–15 min to complete), developed with input from patients, reliable, valid, responsive to change, and is easily scored and interpreted.

There is a growing trend to assess the methodological quality of PROM studies using the Consensus-based Standards for the selection of health status Measurement Instruments (COSMIN) [8]. Systematic reviews on PROMs for specific conditions and symptoms use COSMIN or other assessment criteria to assess which PROMs are superior in terms of validity and other psychometric properties. A core outcome set (COS) is an agreed upon standardized set of outcomes that should be measured and reported, at a minimum, in all clinical trials in specific areas of health or health care. The properties of validated PROMs are being assessed and scientific bodies propose the incorporation of appropriate PROMs into the developed core outcome sets [9]. As part of the uptake of PROMs in practice, the International Consortium for Health Outcomes Measurement (ICHOM), the largest international framework for the collection of PROs, established in 2012, publishes standard sets of outcomes (including PROs) for different medical conditions, together with implementation advice, that hospitals can use to inform what they measure.

PROs can be successfully adopted by clinicians if they fit into the existing ways in which care is organized. Furthermore, PROMs could be used as a means of reorganizing patient care to better meet the needs of patients. The use of computerized-adaptive testing (CAT)-enabled PROMs has expanded over the last decade. The calibration of item banks involves advanced psychometrics using item-response theory. CAT-enabled PROMs are reported to be more individualized than traditional PROMs. The Patient-Reported Outcome Measure Information System (PROMIS), a National Institute of Health initiative, is the most notable example of CAT-enabled PROMs. PROMIS item banks have been created for adult and pediatric populations across physical, mental, and social health. In many cases, items were drawn from other validated PROMs.

Different modes of administration and types of questionnaires can be appropriate for different uses in varying health settings. The recognition of the merits of collecting and reporting PROs and sound knowledge regarding the developmental and psychometric issues related to their optimal use can be the basis of their systematic use towards fostering patient-centered care and sustainable health systems, thus engaging in continuous quality improvement initiatives.
